# VISTA is an activating receptor in human monocytes

**DOI:** 10.1084/jem.20201601

**Published:** 2021-06-09

**Authors:** Bryan M. Rogers, Laura Smith, Zoltan Dezso, Xu Shi, Enrico DiGiammarino, Denny Nguyen, Sunantha Sethuraman, Pingping Zheng, Donghee Choi, Dong Zhang, Andrew Nguyen, Kathleen McGuire, Wei Liu, Namjin Chung, Debra T. Chao, Shiming Ye, Gabriel R. Starbeck-Miller

**Affiliations:** 1 AbbVie Biotherapeutics Inc., Redwood City, CA; 2 AbbVie Inc., North Chicago, IL

## Abstract

As indicated by its name, V-domain Ig suppressor of T cell activation (VISTA) is thought to serve primarily as an inhibitory protein that limits immune responses. VISTA antibodies can dampen the effects of several concomitantly elicited activation signals, including TCR and TLR activation, but it is currently unclear if VISTA agonism could singly affect immune cell biology. In this study, we discovered two novel VISTA antibodies and characterized their effects on human peripheral blood mononuclear cells by scRNA/CITE-seq. Both antibodies appeared to agonize VISTA in an Fc-functional manner to elicit transcriptional and functional changes in monocytes consistent with activation. We also used pentameric VISTA to identify Syndecan-2 and several heparan sulfate proteoglycan synthesis genes as novel regulators of VISTA interactions with monocytic cells, adding further evidence of bidirectional signaling. Together, our study highlights several novel aspects of VISTA biology that have yet to be uncovered in myeloid cells and serves as a foundation for future research.

## Teaser

VISTA biology has yet to be fully resolved despite much enthusiasm to define it as an immune checkpoint protein. Here, we show that VISTA antibodies can singly activate human monocytes and provide evidence that Syndecan-2 can regulate VISTA interactions.

## Introduction

V-domain Ig suppressor of T cell activation (VISTA) is a type I membrane protein with an extracellular IgV-like domain associated with the B7 family (Wang et al., 2011). However, similarities between the extracellular portion of VISTA and the other B7 family members are essentially limited to the presence of the IgV domain (Nowak et al., 2017). One of the most divergent features of the VISTA extracellular domain (ECD) is the C–C′ loop, which contains several residues contributing to a solvent-exposed, positively charged patch (Mehta et al., 2019). Furthermore, the intracellular portion of VISTA is over three times that of its closest relative, programmed death-ligand 1 (PD-L1). Whereas PD-L1 does not contain canonical signaling domains, VISTA contains an SRC homology 2 (SH2) binding motif and three C-terminal SH3 motifs, thus suggesting signaling potential (Nowak et al., 2017). The cytoplasmic domain of VISTA closely resembles the cognate receptors of the B7 family members, the CD28 family (Flies et al., 2011; Nowak et al., 2017). Collectively, the structural elements of VISTA hinted that it can act as a bidirectional signaling molecule because it could function as both a ligand and a receptor.

Early work in VISTA biology indicated that it abrogated immune responses. Genetic ablation of VISTA in mice caused altered frequencies of spontaneously activated T cells and splenic myeloid cells (including monocytes; Wang et al., 2014), which also exhibited increased immune responses to antigenic challenge (Flies et al., 2014; Tang et al., 2010). Immobilized VISTA–Fc (fragment crystallizable domain of IgG1 antibody) fusion proteins were also shown to directly suppress T cell activation in vitro (Gao et al., 2017; Lines et al., 2014; Wang et al., 2014), and recombinant pentameric VISTA suppressed immune responses in vivo (Prodeus et al., 2017). Due to these data and the fact that VISTA is most highly expressed by monocytes and other potential APCs, VISTA was initially proposed to act as a T cell checkpoint inhibitory ligand (Flies et al., 2014; Wang et al., 2011). VISTA was shown to interact with PSGL-1 under acidic conditions and elicited T cell suppression in vitro (Johnston et al., 2019). The authors also observed that pentameric VISTA bound monocytes under physiological pH conditions, suggesting that unknown VISTA ligands could exist on the surface of myeloid cells. This finding in conjunction with prior work strongly argues for an underappreciated role for VISTA biology and regulation on the surface of monocytic cells (Broughton et al., 2019; Han et al., 2019).

Currently, many groups have focused on developing VISTA antibodies that allow therapeutic control of immune responses. Several groups have been able to identify anti-VISTA antibodies with immune-activating (Gao et al., 2017; Johnston et al., 2019; Le Mercier et al., 2014; Liu et al., 2015) and immunosuppressive effects (ElTanbouly et al., 2020; Flies et al., 2011). It has recently been shown that immunosuppressing anti-VISTA antibodies can regulate T cell and innate cell responses when delivered concomitantly with antigen or TLR agonist stimulation, respectively (ElTanbouly et al., 2020; Han et al., 2019). In purified monocytes, immunosuppressive VISTA antibodies were shown to suppress CD14, CD16, and the IFN I pathway (ElTanbouly et al., 2021). Until now, it was unclear if anti-VISTA antibodies could have stand-alone effects on immune cells. In this study, we not only show how VISTA antibodies can singly affect human monocytes as agonists but also identify the monocyte proteoglycan Syndecan-2 (Sdc2) as a novel regulator of VISTA binding to monocytic cells. These results shed critical light on two unexplored facets of VISTA biology and further highlight novel perspectives on the role of this protein outside of T cells.

## Results and discussion

### Novel VISTA antibodies bind unique epitopes of VISTA with high specificity

**Figure 1.**
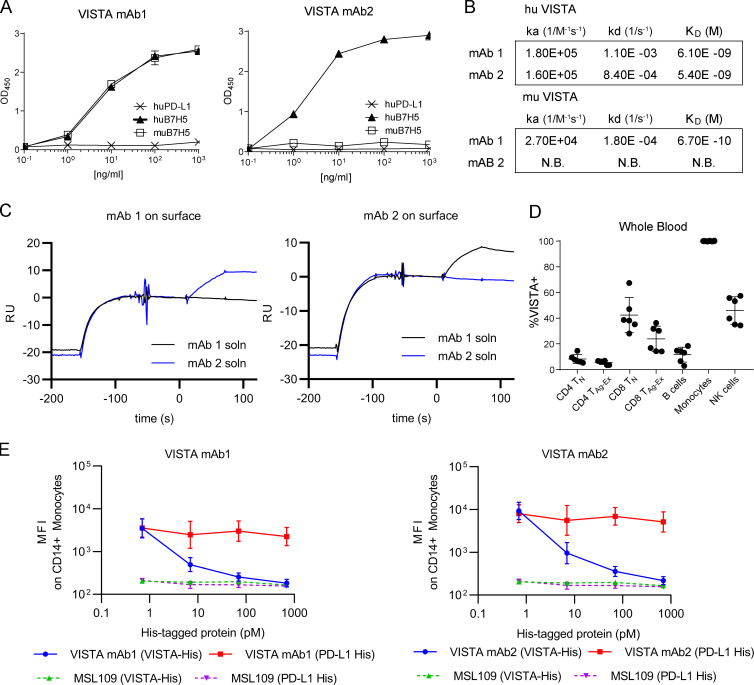
**Binding and specificity of novel anti-VISTA mAbs. (A)** VISTA mAb1 and VISTA mAb2 binding to plate-bound recombinant human (hu) PD-L1, huB7H5, or murine (mu) B7H5 by ELISA. **(B)** Binding kinetics of mAb1 and mAb2 to human and murine VISTA by SPR. No binding (N.B.) was observed for mAb2 to murine VISTA. **(C)** Representative Biacore sensorgram plots from simultaneous binding assay with mAb1 or mAb2 captured on the surface. Antigen binding to surface mAb is shown, followed by injection of solution mAb (soln). RU, reference units. **(D)** Summarized FACS staining results that quantify the percentage of VISTA^+^ cells in human whole blood. *n* = 6 independent donors. T_N_, naive T cells; T_Ag-Ex_, antigen-experienced T cells.** (E)** FACS-based median fluorescence intensity (MFI) of isotype control, mAb1, or mAb2 binding to CD14^+^ PBMCs while in the presence of titrated recombinant His-tagged VISTA (VISTA-His; green triangles or blue circles) or recombinant His-tagged PD-L1 (PD-L1 His; purple triangles or red squares). A–E are representative of three independent experiments.

To more deeply explore the potential of VISTA to serve as a receptor, we conducted anti-VISTA antibody campaigns. Two mAbs that resulted from this effort were named KO11-1B1 (mAb1) and VIBE1A (mAb2). Both mAb1 and mAb2 bound human VISTA while remaining unreactive to its closest homologue, human PD-L1 (Fig. 1 A). Surface plasmon resonance (SPR) analysis revealed that both antibodies bound human VISTA with low nanomolar affinities, which suggested high specificity for the antigen (Fig. 1 B). Because only mAb1 was cross-reactive to murine VISTA (Fig. 1 A), it was not surprising that mAb1 and mAb2 bound unique epitopes of human VISTA, as evidenced by their ability to bind the receptor simultaneously via SPR (Fig. 1 C). FACS staining of peripheral blood mononuclear cells (PBMCs) with mAb1 indicated that B cells, T cells, and natural killer (NK) cells had low to moderate VISTA expression, whereas monocytes exhibited uniformly high expression (Fig. 1 D). The binding of mAb1 and mAb2 during FACS staining was specific because signaling could be quenched by the concomitant addition of excess recombinant VISTA (Fig. 1 E). These data establish that both mAb1 and mAb2 have high affinity and specificity for human VISTA on unique epitopes.

### VISTA mAb treatment alters the transcriptional signature of human monocytes

**Figure 2.**
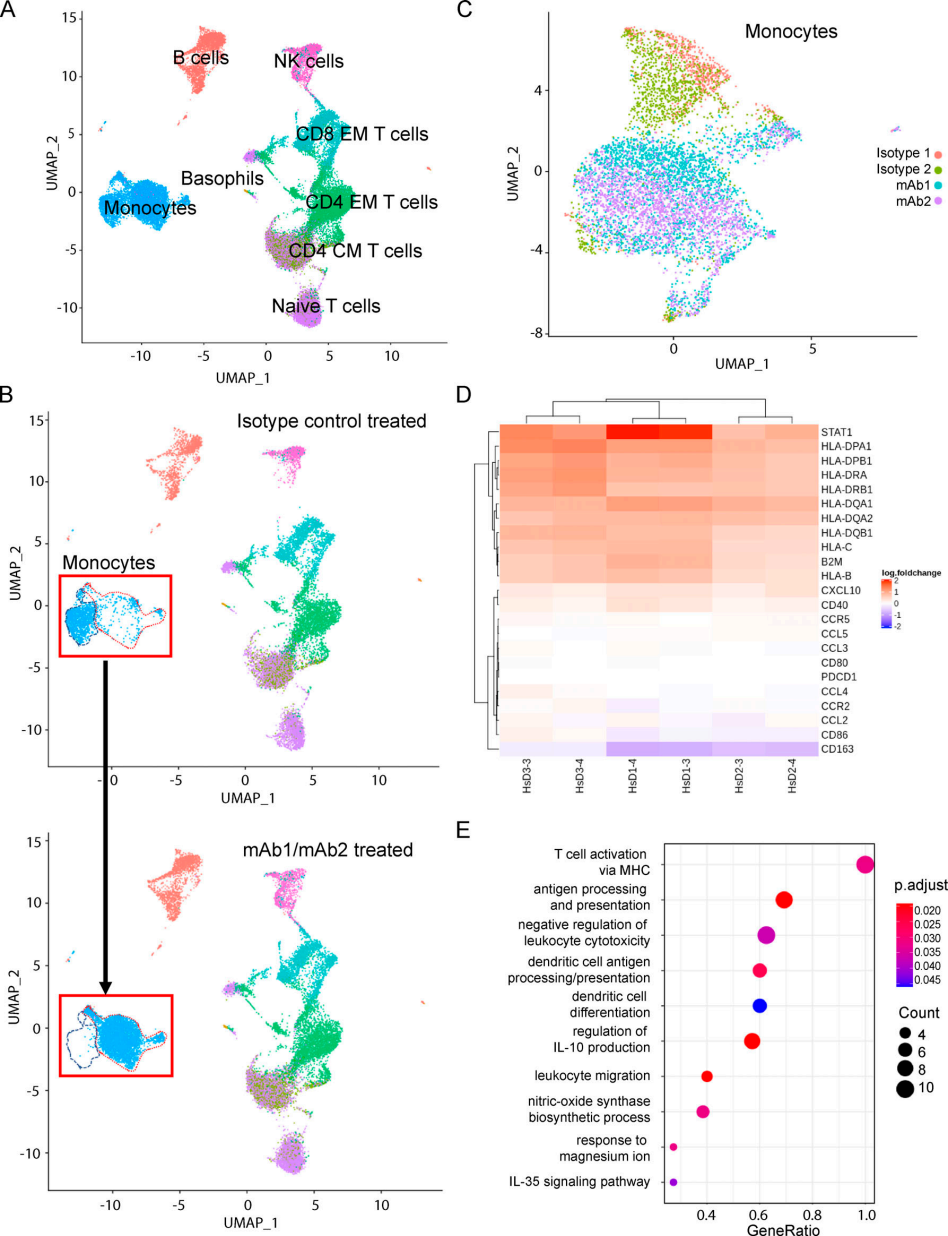
**Monocyte transcriptome is specifically impacted within PBMCs following anti-VISTA antibody treatment.** Human PBMCs were isolated from fresh human whole blood (three donors) and stimulated with either one of two isotype controls (MSL109 or AB095) or one of two anti-VISTA mAbs (mAb1 or mAb2) for 24 h before scRNA-seq/CITE-mAb prep and analyses. **(A)** UMAP of PBMCs from all donors across all treatments. **(B)** Global structural changes of cells between isotype control–treated cells (top) and anti-VISTA mAb–treated cells (bottom). The red dashed line outlines the population of cells in the isotype control treatment group, and the blue dashed line represents the population of cells that were different from the originator population after mAb treatment. **(C)** UMAP clusters of CITE-seq mAb gated monocyte populations after all treatments. **(D)** Heat map of the top gene set enrichment analysis (GSEA) Kyoto Encyclopedia of Genes and Genomes (KEGG) pathway genes that are significantly up-regulated (red) and down-regulated (blue) in CITE-seq mAb monocyte gated PBMCs of three donors (D3, D1, and D2) following anti-VISTA (mAb1 or mAb2) treatment as compared with isotype control (MSL109) treatment. Color represents the expression value of the DEGs (log fold change). **(E)** GSEA of specific KEGG pathways that were activated in both mAb1 and mAb2 treatments. GeneRatio (enriched genes/total number of genes) indicates the extent of pathway activation. The count indicates the number of genes within the pathway and is represented by dot size. The adjusted P values are represented in the color spectrum from 0.020 to 0.045.

**Figure S1.**
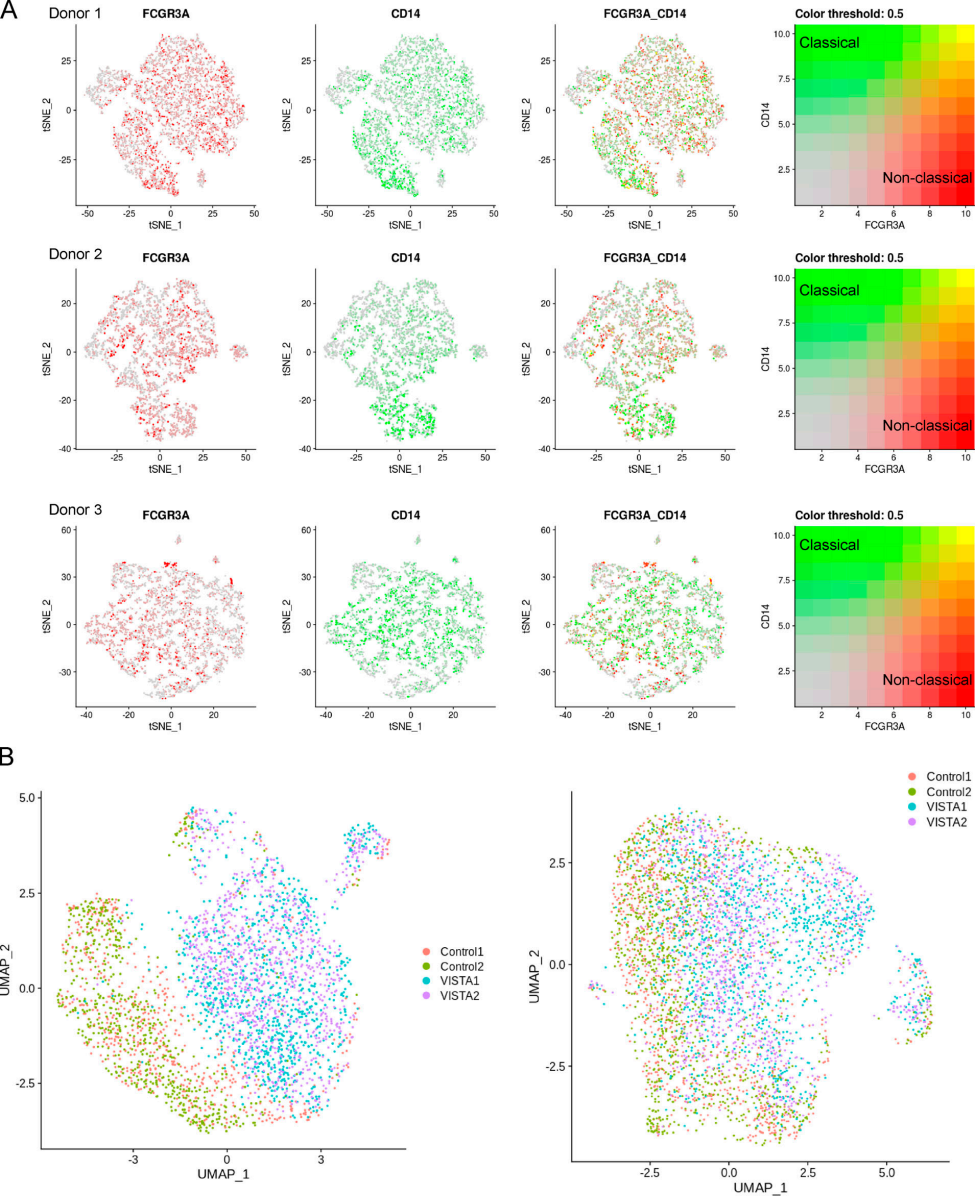
**scRNA/CITE-seq cell markers for monocyte subtyping and two additional donor monocyte clusters.** Related to Fig. 2. **(A)** FcγR3 in red (FCGR3) and CD14 in green were used as subtyping markers for classical (CD14^+^ FCGR3^−^) and nonclassical (CD14^−^ FCGR3^+^) monocytes. Three donors are displayed in the t-distributed stochastic neighbor embedding (tSNE) plots with the representative color threshold for cluster identification. **(B)** Donor 2 (left) and donor 3 (right) UMAP projections of monocytes clustered by anti-VISTA treatment for mAb1 (VISTA1; cyan) and mAb2 (VISTA2; violet). Isotype control treatments are shown as incarnadine and green.

It is not currently known how anti-VISTA antibody treatment can singly affect human PBMC subsets at the level of transcription. To better understand this, we stimulated human PBMCs with our novel anti-VISTA mAb and analyzed downstream effects using single-cell RNA sequencing (scRNA-seq) and Cellular Indexing of Transcriptomes and Epitopes by Sequencing (CITE-seq) experiments (Stoeckius et al., 2017). This analysis enabled the identification of many human PBMC subsets, including monocytes, naive T (TN) cells, CD4 central memory (CM) T cells, CD4 effector memory (EM) T cells, CD8 EM T cells, NK cells, B cells, and basophils (Fig. 2 A). Subtype identification of the monocyte clusters was not possible due to a lack of cluster demarcation between CD14^+^ and CD16^+^ monocyte markers (Fig. S1 A). B cells and T cell subsets did not show broad gene expression changes following VISTA antibody treatment, and CD8^+^ CM T cells were not confidently identified (Table S1). Interestingly, the dimensionality reduction showed that the largest transcriptional changes were in myeloid populations such as NK cells and, more notably, monocytes (Fig. 2 B and Table S1). Since VISTA is most highly expressed by monocytes (Fig. 1 D), and because monocytes demonstrate the broadest transcriptional change following anti-VISTA mAb treatment, we focused our efforts on determining if this was a monocyte-intrinsic effect.

Because the global structure of cells clearly implicated monocytes as being particularly responsive to VISTA antibody exposure, we explored the granularity of gene expression within these populations. Using CITE-Seq–gated CD14^+^ CD3^−^ cells, we clustered gene expression profiles for isotype and VISTA antibody–treated populations (Fig. 2 C and Fig. S1 B). Monocytes clearly clustered by treatment, with isotype-treated cells clustered together and VISTA antibody–treated cells clustered together. These data suggest that both VISTA antibodies may induce common changes in gene transcription in monocytes, despite recognizing unique epitopes. We then performed a gene ontology pathway analysis using the differentially expressed genes (DEGs) between isotype- and VISTA antibody–treated monocytes for both mAbs across all donors (Fig. 2, D and E). VISTA antibody treatments similarly promoted expression of genes involved in antigen presentation/processing (e.g., β_2_-microglobulin, HLA-B, HLA-C, HLA-DRB1, CD68), leukocyte migration (e.g., CXCL9), cell adhesion molecules, dendritic cell differentiation, and cytokine signaling (e.g., STAT1). However, most chemokine/cytokine expression remained relatively unchanged, and the haptoglobin-hemoglobin scavenger receptor, CD163, was down-regulated with both VISTA antibody treatments in two of three donors. Of all the pathways that changed with statistical significance, none were strongly associated with cell death or division (Table S2). Together, these pathway data indicate that the monocyte signature transcriptional shifts observed after VISTA mAb1 and mAb2 treatment in the Uniform Manifold Approximation and Projection (UMAP; Fig. 2 B) dimension reduction technique are likely caused by changes in differentiation status rather than subpopulation expansion and/or contraction.

### Agonistic VISTA mAb treatment elicits monocyte activation in an Fc effector–dependent manner

**Figure 3.**
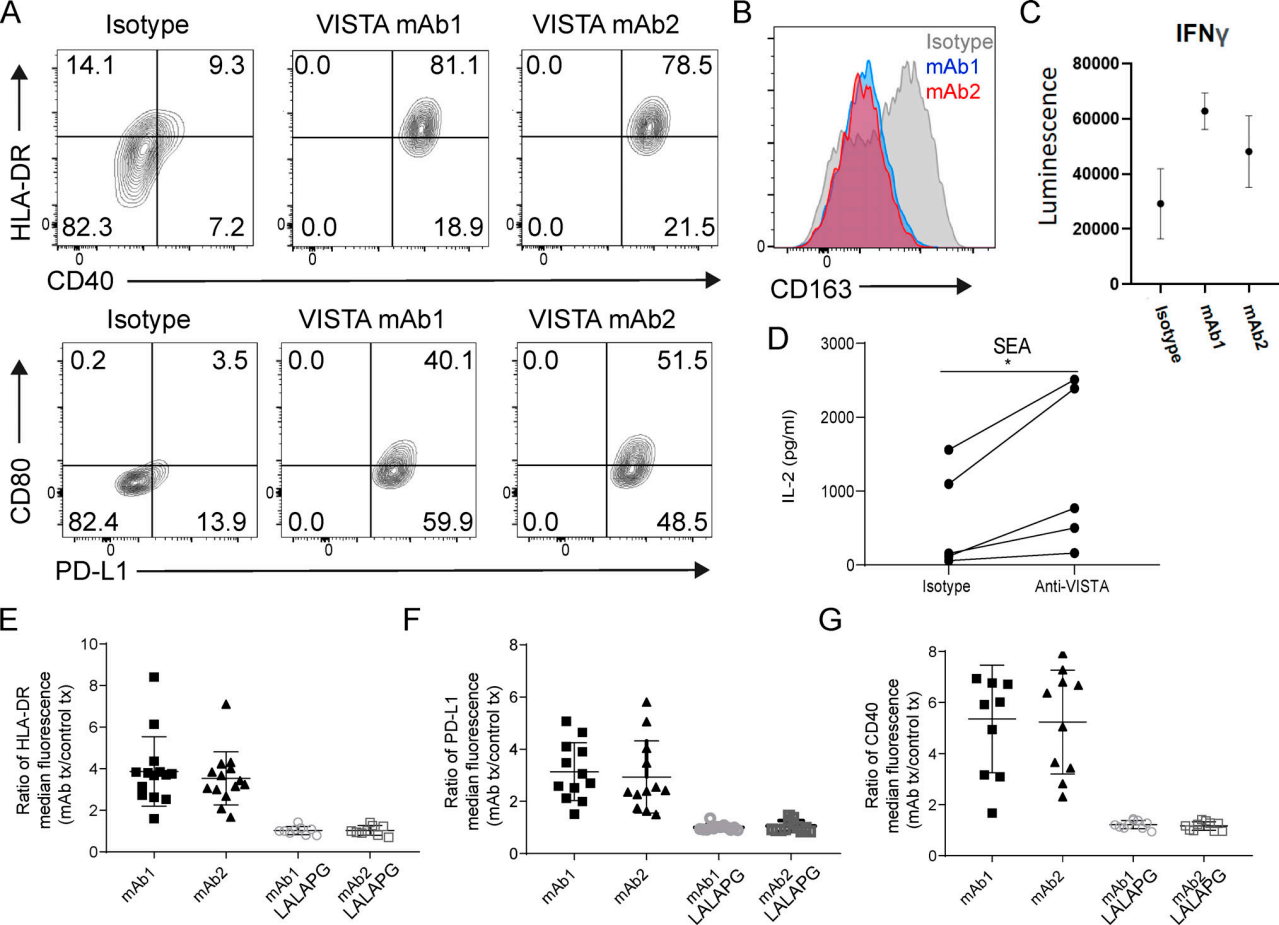
**Monocytes are activated with agonist VISTA antibody treatment in an Fc effector functional manner. (A and B)** Human PBMCs were incubated with either isotype control (MSL109) or VISTA antibody (mAb1 or mAb2) for 24 h, and the monocytes within PBMCs were evaluated for (A) CD40, HLA-DR (top), PD-L1, CD80 (bottom), and (B) CD163 expression by FACS. A and B are representative of >14 independent donors and experiments. **(C)** Human PBMC purified monocytes were incubated for 48 h with mAb1, mAb2, or isotype control antibodies (10 µg/ml) in the absence of stimulation or activation. Human CD4^+^ TN cells were then cocultured with treated monocytes at a ratio of 10:1 in an MLR. Secreted IFNγ was measured after 5 d. **(D) **IL-2 levels in supernatant from human PBMC SEA reaction that was performed with human PBMCs that had been preincubated with either isotype control (MSL109) or anti-VISTA (mAb1) overnight. Student’s *t* test, *n* = 5; *, P < 0.05. C and D were observed after three independent experiments. **(E–G)** Summarized fold increase in (E) HLA-DR (*n* = 14), (F) PD-L1 (*n* = 12), or (G) CD40 (*n* = 9) median fluorescence FACS staining on monocytes within PBMCs following treatment with WT hIgG1 or L234A, L235A, P324G mutations within the Fc domain of human IgG1 (LALAPG) hIgG1 VISTA antibodies (mAb1 or mAb2) relative to appropriately matched isotype controls.

We next sought to confirm that the transcriptional changes elicited by anti-VISTA mAb treatment were manifested at the level of protein expression and function. Treating human PBMCs with mAb1 or mAb2 caused increased surface expression of HLA-DR, CD40, CD80, and PD-L1 with a concomitantly decreased surface expression of CD163 (Mantovani et al., 2013; Stifano and Christmann, 2016) on human CD14^+^ monocytes (Fig. 3, A and B). To better demonstrate that VISTA mAb treatment could sensitize immune responses by engaging monocytes directly, we incubated mAb1 and mAb2 with purified monocytes before introducing them into a mixed-lymphocyte reaction (MLR). VISTA mAb preincubation caused three- and twofold increases of IFNγ secretion from cocultured CD4^+^ T cells (Fig. 3 C). Pretreatment of PBMCs with anti-VISTA antibody before *Staphylococcal* enterotoxin A (SEA)–induced T cell activation resulted in increased IL-2 production, which further demonstrated a functional impact (Fig. 3 D). It was possible that these VISTA mAb effects were driven by either receptor/ligand antagonism or agonism, because the VISTA mAbs were humanized on a WT human IgG1 backbone (Vonderheide and Glennie, 2013). Indeed, the effects were critically dependent on Fcγ receptor (FcγR) interactions because they were lost when mutations (Schlothauer et al., 2016) were introduced to create an Fc-effectorless (L234A, L235A, P324G mutations within the Fc domain of human IgG1) VISTA antibody (Fig. 3, E–G). Due to this effect, it appeared that the anti-VISTA mAb–induced monocyte phenotype was likely not a result of VISTA/VISTA binding partner antagonism or internalization, but rather a result of VISTA receptor cross-linking that was enhanced by Fc-receptor interactions in *cis* or *trans* between myeloid cells. Together, these data suggest that VISTA can serve as an activating receptor on human monocytes and that VISTA antibody ligation can singly alter their functional state.

### VISTA binding to monocytes is modulated by the heparan sulfate proteoglycan Sdc2

**Figure 4.**
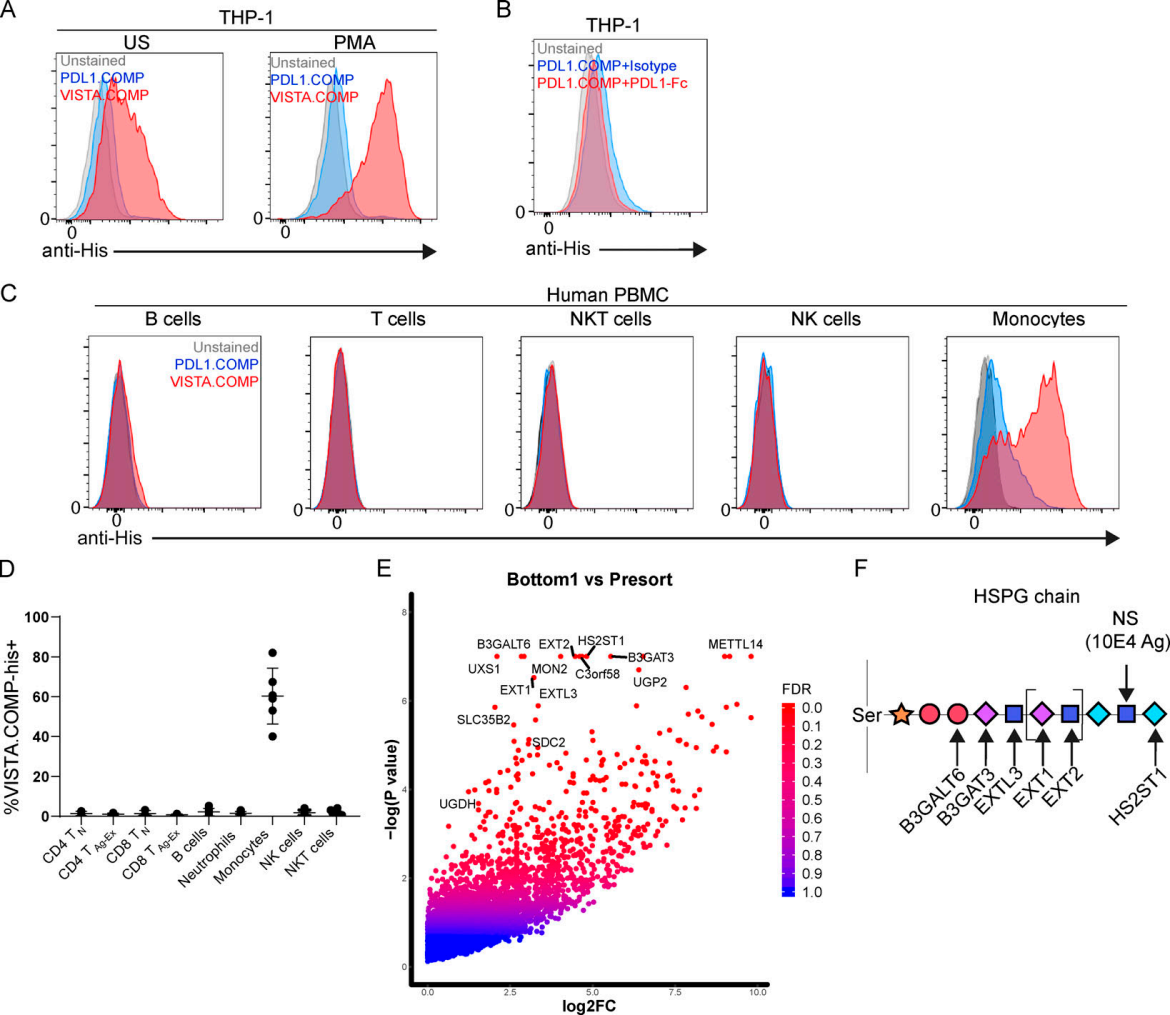
**VISTA oligomer binds to human monocytic cells and identifies Sdc2 and HSPG-related genes as mediators of binding. (A)** Unstimulated (US) or PMA-stimulated THP-1 cells left unstained (gray) or stained with PD-L1.COMP-His (blue) or VISTA.COMP-His (red). Binding was detected by an anti-His secondary antibody. **(B)** PMA-stimulated THP-1 cells left unstained (gray) or stained with PD-L1.COMP (red and blue) in the presence of exogenous isotype control (MSL109; blue) or competing (PD-L1-Fc; red) Fc fusion protein. **(C)** Human PBMCs left unstained (gray) or stained with PD-L1.COMP (blue) or VISTA.COMP (red) protein and markers that identify immune cell subtypes, including B cells, T cells, NK cells, NKT cells, and monocytes. A–C were performed in five independent experiments. **(D)** Summarized results from VISTA.COMP staining of human PBMC subsets from five independent donors. T_N_, naive T cells; T_Ag-Ex_, antigen-experienced T cells.** (E)** Bottom 1% VISTA.COMP^+^ versus pDNA control enrichment of gRNA-targeted genes from CRISPR-based screen expressed as log_2_ fold change (log2FC) by −log(P value) and false discovery rate (FDR). **(F)** A schematic that depicts enzyme hits important for HS chain generation. Xylose (orange star), galactose (red circle), glucuronic acid (purple diamond), *N*-acetyl-D-glucosamine (blue square), and L-iduronic acid (blue diamond). 10E4 mAb epitope (N-sulfated glucosamine [NS]) is also highlighted for reference.

**Figure S2.**
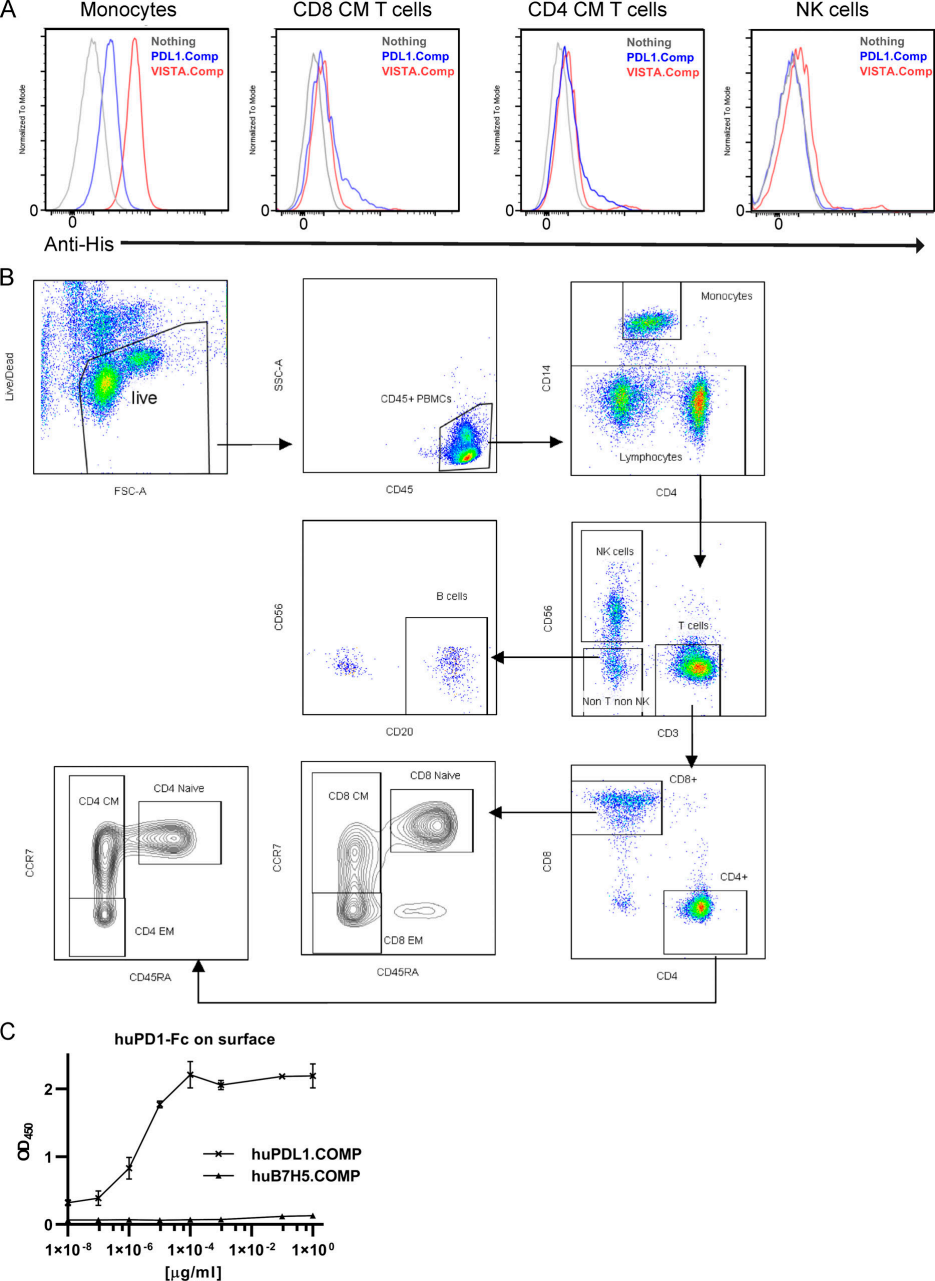
**Characterization of VISTA.COMP and PD-L1.COMP binding on human PBMCs. **Related to Fig. 4.** (A)** Left unstained (gray) or stained with PD-L1.COMP (blue) or VISTA.COMP (red) protein and markers that identify immune cell subtypes, including T cells (CD3^+^), NK cells (CD56^+^), NK T cells (CD3^+^ CD56^+^), and monocytes (CD14^+^SSC^hi^) in the presence of 0.5% paraformaldehyde. FSC, forward scatter; SSC, side scatter. **(B)** Gating strategy for PBMC immune cell identification and T cell subtyping. **(C)** PD-L1.COMP and VISTA.COMP (negative control) binding to plate-bound, recombinant human (hu) PD1-Fc by ELISA. A and C were performed in two independent experiments.

Since VISTA agonism could affect the functional status of human monocytes, we next focused on better understanding the VISTA interactome within a monocyte context. Published data indicate that oligomeric VISTA can bind to monocytes; however, the binding partner was not identified (Johnston et al., 2019). To better understand this interaction, we generated a pentameric VISTA oligomer by fusing a His-tagged cartilage oligomeric matrix protein (COMP) domain to the ECD of VISTA (VISTA.COMP) as done previously (Prodeus et al., 2017). VISTA.COMP was observed to bind resting THP-1 (human monocytic cell line; Auwerx, 1991) with more intensity than unstained or PD-L1.COMP (negative control) conditions, and this effect was magnified following PMA-induced activation (Fig. 4 A). The COMP domain itself did not directly contribute to binding, because PD-L1–Fc fusion protein competition was able to fully quench residual PD-L1.COMP (Fig. 4 B). The weak PD-L1.COMP staining on monocytes was likely due to interactions with CD80/86 costimulatory molecules because they can be expressed by monocytes (Van Gool et al., 1996) and are known PD-L1 binding partners (Butte et al., 2007). The PD-L1 portion of the negative control appeared to be functionally intact and as expected, because it was able to weakly stain PD1^lo^ cell CD4 and CD8 T cell CM subsets (Ribas et al., 2016), and it also bound well to Fc-fused human PD1 (Fig. S2). VISTA.COMP also strongly bound to primary human monocytes and with higher intensity than other PBMC subsets, suggesting that the interaction could be physiologically relevant (Fig. 4, C and D).

### CRISPR/Cas9 screen reveals heparan sulfate proteoglycan (HSPG) pathway enzymes and the proteoglycan Sdc2 as mediators of VISTA binding on monocytes

**Figure S3.**
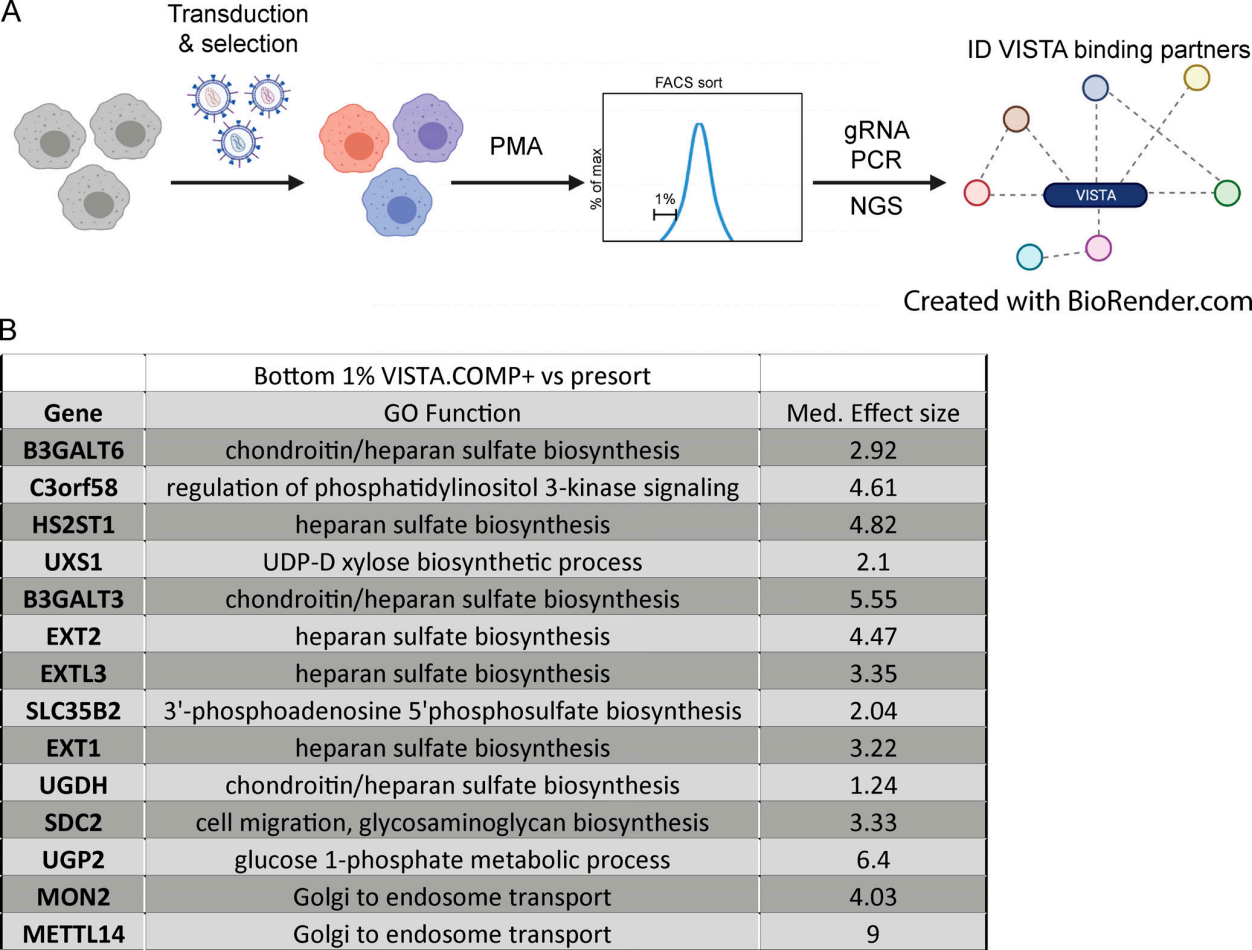
**Genome-wide CRISPR-based VISTA.COMP binding partner screen.** Related to Fig. 4.** (A)** In brief, Cas9-expressing THP-1 cells were transduced with knock-down gRNA-expressing lentivirus, stimulated with PMA, and then stained with VISTA.COMP. The bottom 1% of VISTA.COMP-binding THP-1 were FACS sorted, and their genomic content was subjected to gRNA PCR and NGS in order to assess gRNA enrichment and associated genes that mediate VISTA binding. **(B)** Table of the top genes in the bottom 1% versus before sorting, gene ontology (GO) function, and their relative median effect size.

Using THP-1 cells and VISTA.COMP, we then conducted a genome-wide CRISPR-based identification screen to identify the unknown VISTA binding partner or partners that existed on the surface of monocytes (Fig. S3). The top hits contained many genes (UXS1, B3GALT6, BGALT3, EXTL3, EXT1, EXT2, and HS2ST1) associated with the HSPG generation pathway (Fig. 4 E and Fig. S3). Heparan sulfate (HS) is generated in a particular order by enzymes (Fig. 4 F), similar to a barcode, on core proteins such as glypicans on the cell surface and perlecans in the extracellular matrix. In addition to the identification of HS biosynthesis enzymes, the HS-decorated cell surface proteoglycan Sdc2 (Sarrazin et al., 2011) was also significantly enriched in the bottom 1% of VISTA.COMP^+^ THP-1 cells (Fig. 4 E and Fig. S3). Interestingly, both Sdc2 expression and modification with HSPG (Sdc2-HSPG) have been shown to be critical for monocyte migration, chemotaxis, and maturation (Collins and Troeberg, 2019; Wegrowski et al., 2006). Taken together, these data indicate monocyte-derived Sdc2-HSPG as a novel mediator of VISTA binding to monocytes.

**Figure 5.**
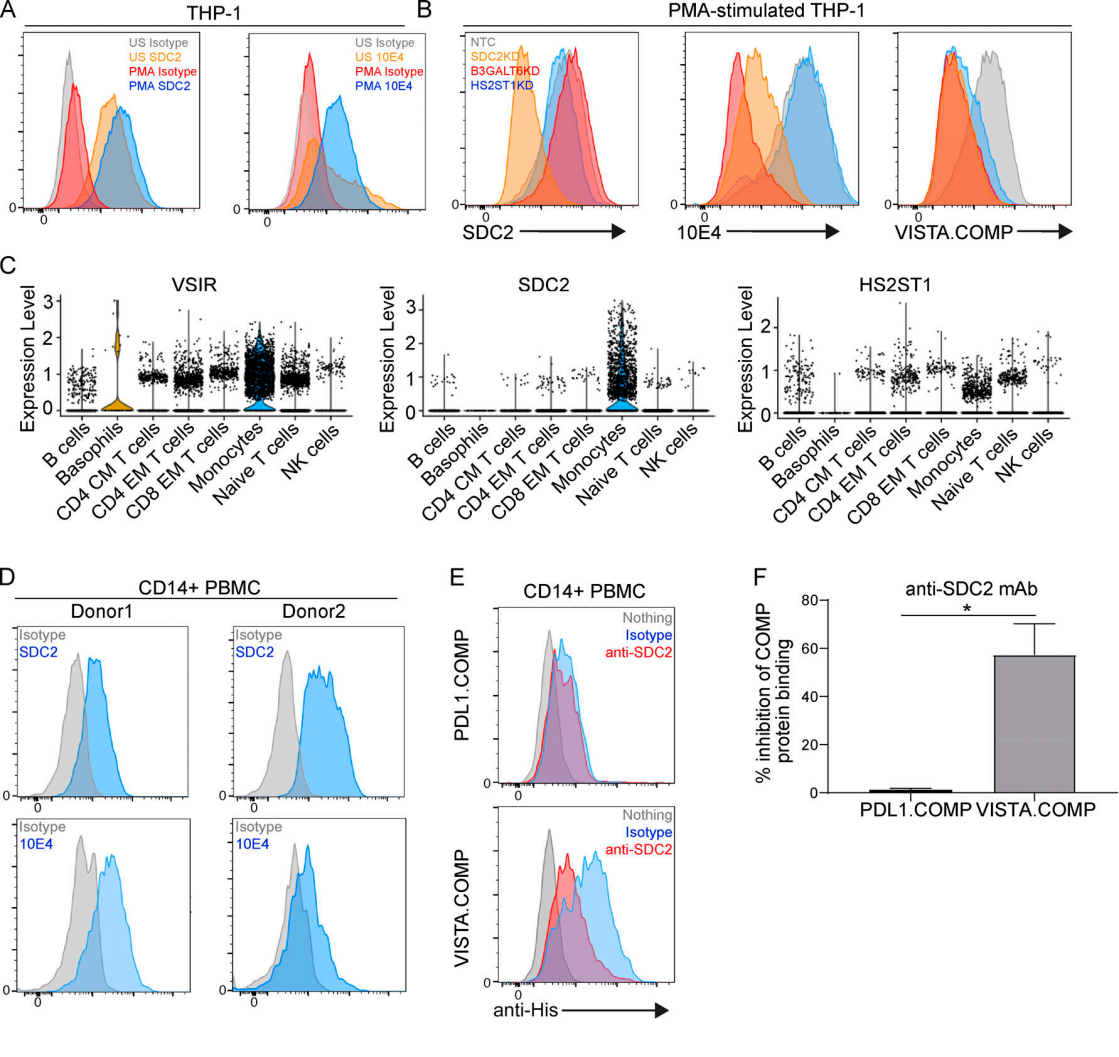
**VISTA binding to human monocytes is dependent on Sdc2-HSPG. (A)** FACS histograms of THP-1 cells that were either left unstimulated (US; gray and orange) or stimulated with PMA (red and blue) before being stained with isotype controls (gray and red), anti-Sdc2 (SDC2; orange and blue; left panel), or 10E4 mAbs (orange and blue; right panel). **(B)** THP-1.CAS9 cells that have been transduced with either a nontargeting (NTC; gray), SDC2 (orange), B3GALT6 (red), or HS2ST1 (blue)-targeting knock-down gRNA and assessed for SDC2 expression (left panel), 10E4 mAb binding (middle panel), and VISTA.COMP (right panel) binding by FACS staining. A and B are representative of three independent experiments. **(C)** Violin plots showing the expression level of VSIR, SDC2, and HS2ST1 by scRNA/CITE-seq for isotype control treatment on human immune PBMCs. **(D)** Human CD14^+^ PBMCs from two independent donors stained with isotype control (gray) or anti-Sdc2 (blue; top row) or 10E4 mAb (bottom row; blue). **(E)** Human CD14^+^ PBMCs left unstained (gray) or stained with PD-L1.COMP (top panel; blue and red) or VISTA.COMP (bottom panel; blue and red) in the presence of isotype control (blue) or Sdc2 antibody (anti-SDC2; red). **(F)** Summarized percentage inhibition of PD-L1.COMP or VISTA.COMP binding in the presence of anti-Sdc2 mAb. *n* = 6 donors; *, P < 0.05. D–F are representative of three independent experiments.

Our initial observations of increased VISTA.COMP binding following PMA stimulation on THP-1 cells indicated that Sdc2 expression and/or its associated HSPG profile could be dynamic and dependent on activation status. However, Sdc2 expression was high on both unstimulated and PMA-activated THP-1 cells (Fig. 5 A). Upon further examination of the potential HSPG profile of Sdc2, N-sulfate glucosamine residue content (mAb 10E4 epitope; Fig. 4 F) was low on resting monocytes but increased substantially following PMA stimulation (Fig. 5 A). CRISPR/Cas9-mediated knock-down of B3GALT6 and HS2ST1 maintained binding of the anti-Sdc2 mAb, and, expectedly, a loss of mAb binding was observed with Sdc2 knock-down (Fig. 5 B, left panel). Knock-down of HS2ST1, and therefore *O*-sulfate content on HSPGs, retained binding of mAb 10E4, given that the epitope encompasses the N-sulfate substituents (van den Born et al., 2005; Fig. 5 B, center panel). Knock-down of Sdc2 substantially decreased 10E4 staining intensity (Fig. 5 B, center panel), thus suggesting that Sdc2 may be a major core protein of N-sulfate HSPG-harboring proteoglycans on monocytic cells. Unsurprisingly, a loss of B3GALT6 (a transferase for the critical proximal stem linkage of HSPGs) caused decreased 10E4 staining (Fig. 5 B, center panel). VISTA.COMP binding to PMA-stimulated THP-1 cells was equivalently dependent on HS chain–elongating enzymes (B3GALT6 and HS2ST1) and Sdc2, supporting their role as critical determinants of VISTA binding in monocytes (Fig. 5 B, right panel). Together, these data indicate that the *O*-sulfate terminal moiety of Sdc2-HSPG likely mediates interactions to VISTA. Indeed, a review of our scRNA/CITE-seq data for the isotype control treatments on PBMCs revealed that Sdc2 and the HSPG enzyme HS2ST1 were expressed by monocytes (Fig. 5 C). Both Sdc2 and N-sulfated glucosamine (10E4 epitope) were also detected by FACS (Fig. 5 D), in agreement with our earlier data of VISTA.COMP binding to CD14^+^ PBMCs (Fig. 3 C). Importantly, an Sdc2 antibody inhibited the ability of VISTA.COMP to bind to primary human monocytes while not affecting PD-L1.COMP binding (Fig. 5, E and F). Collectively, these data further confirm that Sdc2-HSPG is a binding mediator of VISTA on monocytes.

It was recently shown that “agonistic” anti-VISTA mAbs can bind VISTA on the surface of T cells to promote antigen-induced tolerance (ElTanbouly et al., 2020) and bind VISTA on the surface of innate immune cells (plasmacytoid dendritic cells and neutrophils) in order to inhibit TLR responses (Han et al., 2019). Until now, it was unclear if agonistic VISTA antibodies can singly affect human immune cell biology. We show that two VISTA antibodies increase human monocyte expression of surface proteins that regulate antigen presentation (e.g., HLA), T cell costimulation (e.g., CD80 and PD-L1), APC maturation (CD40), and potential to activate T cells. Because these effects required intact Fc-receptor binding and could be equally elicited by antibodies of two independent specificities, we suspect this mAb-mediated phenotype requires VISTA receptor cross-linking and downstream agonistic signaling. The scRNA-seq data for monocytes did not reveal significant changes in the FcγR pathway. In light of recently published data (ElTanbouly et al., 2020; Han et al., 2019), our study suggests that the downstream immune-suppressive or -activating consequences of VISTA agonism may be dependent on the immune cell subset and/or concomitant signaling context (e.g., VISTA agonism alone or in combination with TCR or TLR) that may interact with or be influenced by VISTA-mediated signaling pathways in a bidirectional receptor signaling mechanism.

Deficiency of VISTA in macrophages and myeloid-derived suppressor cells has been shown to induce chemotactic paralysis through an elevation of the chemokines CCL3, CCL5, and CXCL10 by an unknown mechanism (Broughton et al., 2019). The authors proposed several possible mechanisms that may govern this phenomenon; however, syndecans or other HSPG core proteins were not considered. HSPGs are known to bind chemokines directly for immobilizing and retaining chemokines to the surface of endothelial cells to promote chemotactic migration of immune cells (Collins and Troeberg, 2019; Sarrazin et al., 2011). HSPG can also serve to promote cell surface retention of MCP-1 (CCL2), MIP-1α (CCL3), and Rantes (CCL5) to influence their *cis* interaction with cognate chemokine receptors on the same cell (Hoogewerf et al., 1997). It stands to reason that VISTA interactions with Sdc2-HSPG may regulate monocyte biology through chemokine gradients and/or signaling. While the exact HSPG barcode remains undeciphered, a list of critical enzymes involved in Sdc2-HSPG specificity for VISTA are clearly defined. Data from the Korman group suggested that Chinese hamster ovary cell–specific HSPG could interact with VISTA (Johnston et al., 2019); however, our work outlines a more defined and physiologically relevant context that establishes the groundwork for further exploration into the importance of these interactions.

## Materials and methods

### Subjects and samples

The study was approved by the institutional review board at AbbVie Biotherapeutics. Human blood samples were collected from healthy donors who registered for the AbbVie Biotherapeutics Employee Blood Collection Program in Redwood City, CA. Whole blood was obtained in heparin-anticoagulated tubes (BD Biosciences) and then processed for staining on the day of collection.

### Media, chemicals, and recombinant protein production

RPMI (HyClone) medium was commonly used and contained 10% heat-inactivated FBS, penicillin/streptomycin (Lonza), and GlutaMAX (HyClone). PMA (P1585) and purified HSPG (H4777) were purchased from Sigma-Aldrich. DNA encoding the full-length human and murine VISTA, human PD-L1, human antibody heavy chains, and human light chains were separately cloned (with a C-terminal COMP domain and His-tag or untagged Fc for fusion proteins) into a modified mammalian pHybE expression vector with their respective signal peptides. A pHybE vector was chosen because it contains the EBV origin of replication for higher-yield expression of dual-expression vectors as described previously (Gu and Ghayur, 2012). Expression and transfection were performed by transiently transfecting HEK293–EBV nuclear antigen cells cultured in 293 Freestyle Media with a 3:2 ratio of light chain to heavy chain vector and a 4:1 ratio of polyethylenimine to DNA. Expression was allowed to proceed for 7 d at 37°C and 8% CO_2_. The secreted proteins were isolated from cells by depth filtration using an AcroPak 1000 capsule. Recombinant proteins with a His-tag were captured by immobilized metal affinity chromatography using HisTrap excel columns on an ÄKTA pure 25 system. The columns were equilibrated with 5 column volumes (CVs) of 50 mM Tris, pH 7.6, with 300 mM NaCl (buffer A). Following capture, the columns were washed with 5 CVs of 90% buffer A and 10% 50 mM Tris, pH 7.6, 50 mM NaCl, and 400 mM imidazole (buffer B). Elution was performed with a 20-CV gradient from 10% to 100% buffer B. The proteins were further purified by size exclusion chromatography using a Superdex 200 26/600 prep grade column equilibrated in PBS, pH 7.4. Proteins with an Fc were captured by affinity chromatography on MabSelect SuRe resin. The columns were equilibrated and washed with 5 CVs of PBS, pH 7.4, and the elution was performed with 5 CVs of 50 mM glycine, pH 3.0. The proteins were then buffer exchanged into PBS, pH 7.4, by tangential flow filtration. Confirmation of identity and post-translational modifications were performed by reduced and intact liquid chromatography–mass spectrometry on a Waters Xevo G2-XS QTof with Bio I class ultra-performance liquid chromatography by UNIFI software running a Waters C4 reversed-phase column in 0.1% formic acid mobile phases of water and acetonitrile. Purity was verified by PAGE of reduced and intact samples. Native fold and aggregate content was assessed using Bio I class ultra-performance liquid chromatography running PBS through a BEH 200 size exclusion chromatography column.

### FACS/ELISA reagents and antibodies

Buffer used for FACS staining was a PBS, pH 7.2 (HyClone)–based 1% BSA (Rockland Immunochemicals) 1 mM EDTA (Teknova) solution. Fc block was made by diluting heat-inactivated human AB serum (Valley Biomedical) to a 20% solution in FACS buffer. CD163 (GHI/61), HLA-DR (L243), CD40 (5C3), PD-L1 (29E.2A3), CD80 (2D10), CD14 (63D3), CD3 (OKT3), CD45RO (UCHL1), CD19 (4G7), CD56 (5.1H11), CD11b (M1/70), streptavidin-PE (405204), anti-mouse IgM PE (RMM-1), and anti-His PE (J095G46) FACS antibodies were all purchased from BioLegend. Sdc2 antibody (305515) was purchased from R&D Systems. Anti-HS antibody 10E4 (F58-10E4) was purchased from AMSBIO. CITE-seq mAbs (TotalSeq B) included CD80 (2D10), CD274 (29E.2A3), CD3 (UCHT1), CD19 (HIB19), CD45RA (HI100), CD4 (RPA-T4), CD8 (RPA-T8), CD14 (M5E2), CD16 (3G8), CD56 (QA17A16), CD335 (9E2), CD62L (DREG-56), CD197 (G043H7), HLA-DR (L243), CD11b (ICRF44), and CD45RO (UCHL1; Table S4). Streptavidin-HRP (016-030-084) was purchased from Jackson ImmunoResearch Laboratories. The ELISA Substrate Reagent Pack (DY999), ELISA plate-coating buffer (DY006), reagent diluent concentrate 2 (DY995), and Stop Solution (DY994) were purchased from R&D Systems. Anti-VISTA mAb1 and mAb2 were generated using standard hybridoma technology by immunizing mice with either mouse VISTA-Fc fusion proteins (mAb1) or human VISTA-expressing CHOK1 cells and boosted with human VISTA-Fc (mAb2). Isotype 1 (Iso1) and isotype 2 (Iso2) were specificities belonging to MSL-109 (anti-CMV) and AB095 (anti-tetanus toxoid) and were engineered on a human IgG1 backbone.

### Binding kinetics and epitope grouping (competition) studies

Binding kinetics and epitope grouping (competition) studies of anti-VISTA were determined by SPR using a Biacore T200 instrument (GE Healthcare). Studies were performed in HBS-EP+ buffer (10 mmol/liter HEPES, pH 7.4, 150 mmol/liter NaCl, 3 mmol/liter EDTA, 0.05% Tween 20). A capture chip was prepared with ∼2,000 reference units of goat anti-mouse or anti-rat IgG Fc polyclonal mAb (Thermo Fisher Scientific), immobilized across a CM5 biosensor chip using a standard amine coupling protocol. Recombinant human and mouse VISTA ECD proteins (His-tags) were produced in-house (HEK293).

Binding kinetics were assayed at 25°C, and each cycle consisted of the following steps: (1) capture of test mAb on test surface only; (2) analyte injection (VISTA ECD or buffer only) over both reference and test surfaces, after which the dissociation was monitored; and (3) regeneration of capture surface by low-pH glycine. Analyte injections were randomized five-point, threefold dilution series from a 900 nM top dose. During the assay, all measurements were referenced against the capture surface alone (i.e., with no captured test antibody), and buffer-only injections were used for secondary referencing. Data were processed and fitted globally to a 1:1 binding model using Biacore T200 evaluation software to determine the binding kinetic rate constants, *k*_a_ (M^−1^ s^−1^) and *k*_d_ (s^−1^), and the equilibrium dissociation constant *K*_D_ (M).

Epitope grouping (competition) studies were performed at 12°C to slow the off rate of the interaction. Each assay cycle consisted of the following steps: (1) capture of the first test mAb at 10 μg/ml on test surface only, and all other injections were over both reference and test surfaces; (2) blocking injection of isotype control cocktail at 100 μg/ml; (3) analyte injection; (4) second test mAb injection at 10 μg/ml; and (5) regeneration of capture surface by low-pH glycine.

### PBMCs and THP-1 culture

Peripheral whole blood was harvested in K2 EDTA tubes. PBMCs were separated from whole blood by density gradient centrifugation using Ficoll-Paque (GE Healthcare) and Leucosep tubes (Greiner Bio-One GmbH) according to the manufacturer’s protocol (MACS; Miltenyi Biotec GmbH). After PBMC isolation, cells were either stimulated with antibodies or rested overnight before COMP fusion protein/FACS staining. For antibody stimulations, fresh PBMCs were incubated with 20 μg/ml antibody overnight before harvest and FACS staining. THP-1 cells were cultured in RPMI-based media at a density ∼2.5 × 10^5^/ml before 5 ng/ml PMA stimulation where indicated. After 48 h, cells were detached with enzyme-free dissociation buffer before the FACS staining protocol. Gating of cells was performed with the following strategy: CD4 T cells (CD4 TN; CD3^+^CD4^+^CD45RO^−^), antigen-experienced CD4 T cells (CD3^+^CD4^+^CD45RO^+^), CD8 TN cells (CD3^+^CD8^+^CD45RO^−^), antigen-experienced CD8 T cells (CD3^+^CD8^+^CD45RO^+^), B cells (CD3^−^CD14^−^CD19^+^), monocytes (high side scatter; CD3^−^CD19^−^CD14^+^), or NK cells (CD3^−^CD19^−^CD14^−^CD56^+^) in human whole blood.

### T cell stimulation assay

Pretreated PBMCs with anti-VISTA antibody were stimulated with 100 ng/ml SEA peptide (Toxin Technology, Inc.) for 4 d at 37°C, 5% CO_2_. After the incubation, cell-free supernatants were harvested, and IL-2 cytokine levels were quantified using AlphaLISA (PerkinElmer) according to the manufacturer’s protocol.

### MLR assay

Monocytes were purified from fresh human blood. Briefly, human PBMCs were isolated using a Ficoll gradient and allowed to adhere to the plate for 2 h, after which cells in suspension were removed. Fresh AIM V medium (Thermo Fisher Scientific) was used without activation or stimulation reagents. mAb1, mAb2, or isotype control antibodies were separately incubated with monocytes at 10 µg/ml for 48 h. Treated monocytes were then cocultured with viably thawed CD4 T cells (Biological Specialty Corporation) at a ratio of 10:1 (T cells to monocyte-derived dendritic cells) in an MLR. The MLR was cultured for 5 d, after which the cells were analyzed by flow cytometry using an LSRFortessa X-20 instrument (BD Biosciences) to determine cell numbers and functional cytokine (IFNγ) responses. Secreted IFNγ was analyzed using a human IFNγ AlphaLISAγ Detection Kit per the manufacturer’s recommendations (PerkinElmer).

### CITE-seq antibody staining and scRNA-seq

Stimulations were done with either one of two human IgG1 isotype controls (Iso1 and Iso2) or novel anti-VISTA mAbs (mAb1 and mAb2, human IgG1 backbone framework) to control for unforeseen specific isotype control effects and to explore possible epitope-dependent nuances between mAb1- and mAb2-induced biology, respectively. This analysis revealed 21 cell clusters, of which 5 were myeloid cells and 16 were lymphocytes, and further delineation was performed using both scRNA-seq and CITE-seq datasets. TN, CM, and EM T cell subtype identifications were delineated with the CD3, CD4, CD8, CD45RA, and CD197 canonical markers from TotalSeq B antibodies using the CITE-seq data across all clusters. Dimension reduction of all treatments across all donors was performed using UMAP (McInnes et al., 2018 *Preprint*) with both scRNA-seq and CITE-seq datasets for T cell subtype analysis and all cell clusters according to the manufacturer’s protocol (https://www.biolegend.com/en-us/protocols/totalseq-b-or-c-with-10x-feature-barcoding-technology). Briefly, a total of 1–2 million cells in 50 µl Cell Staining Buffer (BioLegend; catalog no. 420201) were used for antibody staining. After adding 5 µl Human TruStain FcX blocking reagent (BioLegend; catalog no. 422301) and incubating for 10 min at 4°C, antibody cocktail with 1 µg of each TotalSeq B antibody (Table S4) was added to the cell suspension to bring the total volume to 100 μl. Incubation was allowed to proceed for 30 min at 4°C for antibody staining. 3.5 ml of cell staining buffer was used to wash cells three times. Cells were resuspended in PBS with 0.04% BSA (Thermo Fisher Scientific; catalog no. AM2618) to a concentration of 1 × 10^6^ cells/ml. Following the manufacturer’s protocol (Chromium Single Cell 3′ V3 Chemistry; 10X Genomics), a single-cell suspension with RT-PCR master mix was loaded into the Chip B and Chromium Controller. Single cells with barcoded gel beads were encapsulated into nanoliter-sized Gel Bead-in-Emulsion. After performing RT-PCR within the Gel Bead-in-Emulsion, each transcript was barcoded and pooled for cDNA enrichment. The libraries were prepared using amplified cDNAs for next-generation sequencing (NGS; Illumina sequencing by synthesis chemistry). Each cell had, on average, 80,000 sequencing reads (Table S5). Raw fastq data of each sample were processed by cellranger count (version 3.0.2) following the manufacturer’s protocol. The VISTA antibody–treated PBMCs were aggregated with isotype-treated samples from the same donor using the cellranger aggr (version 3.0.2) function. The cloupe files generated from the cellranger aggregation results were viewed and further analyzed in the Loupe Cell Browser. The distributions of CD3 and CD14 mAb values showed a clear bivariate normal distribution. To define the monocytes, we fitted a two-component mixture model using the function “normalmixEM” from the “mixtools” package in R. We determined the threshold values where cells had 0.5 probability of belonging to either of the two normal distributions (+ or −). Based on these threshold values, cells were assigned to the CD3^+/−^ and CD14^+/−^ groups, respectively. The cells assigned to both the CD3^−^ and CD14^+^ groups were annotated as monocytes. The rank sum Wilcoxon test was performed (as implemented in R) between the treatment and isotype groups in monocytes to identify DEGs in monocytes across all donors following mAb1 or mAb2 treatment. A fold-change value of 1.5 and an adjusted P value of 0.1 were used to determine the set of significantly altered genes under the treatments (Table S1). Data are available through the Gene Expression Omnibus database under accession GSE173747.

### THP-1 Cas9 cell line generation

THP-1 cells were transduced with a lentivirus that stably expresses Cas9 protein once integrated into the host genome and selected for integration with 7.5 μg/ml blasticidin. The Cas9 activity of the cells was characterized through GFP disruption assay and determined to be >80%.

### CRISPRx screen in THP-1 Cas9 monocyte cell line

THP-1 Cas9 cells were transduced with the Brunello genome-wide library (Broad Institute) at MOI 0.3 with 1:1,000 LentiBlast reagent (catalog no. LBPX1500) and 8 μg/ml polybrene (TR-1003-G) by spinfection for 1 h at 200 ×*g* at room temperature. Transduction was performed in triplicate at 500× coverage, and cells were replated at 1e^6^/ml in RPMI, 10% FBS, 1% penicillin/streptomycin, and 7.5 μg/ml blasticidin. After 1 d, transduced cells were selected with 1.5 μg/ml puromycin for 4 d before dead-cell removal with the Miltenyi Dead Cell Removal Kit (catalog no. 130-090-101) as per the manufacturer’s protocol. Cells were allowed to expand for 6 d before differentiation with PMA at 4 ng/ml for 48 h. Cells were harvested using HBSS-based enzyme-free dissociation buffer (EMD Millipore; S-004-C). 40e^6^ cells per replicate were kept as presort controls before VISTA.COMP staining protocols. Cells were stained at 5e^6^/ml via Fc block with 20% heat-inactivated human AB serum in FACS buffer (Valley Biomedical; HP1022H1) as previously described, before a 1-h stain with VISTA.COMP.His protein or VISTA.His control. Cells were then secondarily stained with anti-His PE followed by a 1:1,000 Zombie Violet live/dead stain, then fixed with 1% paraformaldehyde for 1 h at 4°C. Cells were washed with FACS buffer and stored overnight before being sorted with a BD FACSAria cell sorter. Control VISTA.His cells were run to establish gating of VISTA.COMP.His-stained cells. Cells were sorted into the bottom 1% VISTA.COMP.His binders at numbers needed to maintain 500× coverage per replicate, washed, and frozen down before genomic DNA (gDNA) extraction and submission for NGS.

### CRISPR screen NGS

NGS amplicon library preparation was performed using a single-step PCR in 100-µl PCRs for 28 cycles using ≤10 µg gDNA per reaction using Takara/Clontech Titanium Taq DNA Polymerase and PCR buffer (catalog no. 639242) and Takara/Clontech dNTPs (catalog no. 4030). When >10 µg gDNA was available for a given sample condition, the entire quantity of gDNA was used for NGS amplicon library preparation by setting up multiple 100-µl PCRs and then combining post-PCR amplicon reactions from each respective sample before solid-phase reversible immobilization bead cleanup and quantitation. All NGS amplicon libraries were pooled and sequenced together for 75 cycles and demultiplexed using an 8-bp single-ended index barcode incorporated during the PCR.

### CRISPR screen data analysis

Guide sequences were counted from sequencing reads using a custom Perl script. Only reads with exact matches to the guide sequence were counted. Screen and sample quality were assessed based on alignment percentage, library representation in sequenced reads, and replicate correlation, and only samples that passed quality control checks were retained for further analysis. Guide counts were normalized using the trimmed mean of M values normalization method from the edgeR R package (Robinson and Oshlack, 2010; McCarthy et al., 2012). Guide-level enrichment/depletion was calculated using the limma-voom method from the R package limma (Law et al., 2014). Gene-level enrichment/depletion was calculated using an internal R-based implementation of the robust rank algorithm (Kolde et al., 2012). Genes with a false discovery rate <0.1 were deemed significant (Table S3).

### COMP fusion protein and FACS staining

Between 2 × 10^5^ and 2 × 10^6^ cells were incubated with 100 μl Fc block (20% human AB serum diluted in FACS buffer) for 30 min at 4°C. Cells were washed twice before being left unstained or stained with the specified COMP-His fusion protein (30 μg/ml or ∼250 μM diluted in FACS buffer) for 1 h at 4°C. Cells were then washed twice before being introduced to primary FACS antibodies and anti-His secondary antibodies for 30 min at 4°C. Cells were then washed twice before resuspension and FACS analysis. Staining involving coincubation of PD-L1.COMP and PD-L1-Fc was performed at a molar ratio of 1:20, respectively. Blocking of PD-L1.COMP and VISTA.COMP stains was also performed by concomitantly adding 20 μg/ml purified HSPG or anti-Sdc2 antibody.

### ELISA

Antibodies were coated on nontreated 96-well plates at a concentration of 10 μg/ml (PD-1–Fc incubated at 0.1 μg/ml) in ELISA plate-coating buffer overnight at room temperature. Wells were washed three times with PBS before blocking with 1× reagent diluent 2 per the manufacturer’s recommendations. Diluent was decanted, and then wells were incubated with titrated amounts of recombinant protein for 2 h at room temperature. Wells were then washed three times with 1× reagent diluent 2 before incubation with streptavidin-HRP (or HRP–anti-His antibody for COMPs) per the manufacturer’s recommendations. Wells were then washed three times and blotted dry before they were incubated with ELISA Substrate and Stop Solution. The OD of samples was read at a wavelength of 450 nm.

### Online supplemental material

Fig. S1 pertains to the scRNA/CITE-seq data and depicts the monocyte subtype markers CD16 (FcγRIII) and CD14, which did not cluster, and two additional donors for the monocyte-gated transcriptional changes upon VISTA mAb treatment compared with an isotype control. Fig. S2 shows VISTA.COMP and PD-L1.COMP binding to PBMC immune cells, the gating strategy used for determination of immune cells and T cell subtypes, and an ELISA of PD-L1.COMP and VISTA.COMP binding to plate-bound PD1–human IgG1 Fc. Fig. S3 contains an outline of the experimental summary for the CRISPR/Cas9 genome-wide KO screen and the top gene hits in the bottom 1% versus before sorting. Table S1 contains the full list of statistically significant transcriptional changes of all genes for VISTA mAb treatment compared with an isotype control for all identified cell types in PBMCs. Table S2 lists all pathways identified by the DEGs that underwent changes upon VISTA mAb treatment compared with an isotype control. Table S3 contains all genes in the CRISPR/Cas9 KO screen that were shown to affect VISTA.COMP binding in the bottom 1% versus before sorting. Table S4 lists the TotalSeq antibodies used in the CITE-seq clustering of PBMCs between isotype and VISTA treatments. Table S5 summarizes the scRNA-seq data quality and depth.

## Supplementary Material

Table S1 lists genes found to be differentially expressed compared with isotype for all cell types.

Table S2 lists GSEA pathways identified to be differentially enriched compared with isotype for monocytes.

Table S3 lists gene hits in the bottom 1% versus before sorting from CRISPR/Cas9 KO screen.

Table S4 lists CITE-seq antibodies used for cell clustering analysis.

Table S5 shows quality control and data metrics on the sequencing depth and quality.
